# Electrophysiological properties of mouse and epitope-tagged human cardiac sodium channel Na _v_1.5 expressed in HEK293 cells

**DOI:** 10.12688/f1000research.2-48.v2

**Published:** 2013-04-05

**Authors:** Katja Reinhard, Jean-Sébastien Rougier, Jakob Ogrodnik, Hugues Abriel

**Affiliations:** 1 Department of Clinical Research, University of Bern, Bern, 3010, Switzerland; 2 Current address: Centre for Integrative Neuroscience, University of Tübingen, Tübingen, 72076, Germany

**Keywords:** Cardiac sodium channel, Nav1.5, HEK293 cells, electrophysiology

## Abstract

**Background: **The pore-forming subunit of the cardiac sodium channel, Na _v_1.5, has been previously found to be mutated in genetically determined arrhythmias. Na _v_1.5 associates with many proteins that regulate its function and cellular localisation. In order to identify more *in situ* Na _v_1.5 interacting proteins, genetically-modified mice with a high-affinity epitope in the sequence of Na _v_1.5 can be generated.

**Methods:** In this short study, we (1) compared the biophysical properties of the sodium current (I _Na_) generated by the mouse Na _v_1.5 (mNa _v_1.5) and human Na _v_1.5 (hNa _v_1.5) constructs that were expressed in HEK293 cells, and (2) investigated the possible alterations of the biophysical properties of the human Na _v_1.5 construct that was modified with specific epitopes.

**Results: **The biophysical properties of mNa _v_1.5 were similar to the human homolog. Addition of epitopes either up-stream of the N-terminus of hNa _v_1.5 or in the extracellular loop between the S5 and S6 transmembrane segments of domain 1, significantly decreased the amount of I _Na_ and slightly altered its biophysical properties. Adding green fluorescent protein (GFP) to the N-terminus did not modify any of the measured biophysical properties of hNa _v_1.5.

**Conclusions:** These findings have to be taken into account when planning to generate genetically-modified mouse models that harbour specific epitopes in the gene encoding mNa _v_1.5.

## Introduction

The voltage-gated cardiac sodium channel Na _v_1.5 is responsible for the initial phase of the cardiac action potential and plays a central role in cardiac impulse propagation ^1^. Its role in human disorders has been underlined by the findings of several hundred mutations in its gene, *SCN5A*, that are linked to inherited cardiac electrical disorders such as congenital long QT syndrome and Brugada syndrome ^2^. In recent years, it has been demonstrated that Na _v_1.5 interacts with and is regulated by different proteins (recently reviewed by Shy *et al.* ^3^). Many of these interacting proteins were also found to be mutated in patients with genetically-determined cardiac arrhythmias ^4^. The generation of genetically-modified mouse models, harbouring mutations in the *Scn5a* gene, has proven to be a very informative approach to investigate the various human phenotypes that are linked to the genetic variants of this gene ^5^. Since Na _v_1.5 interacts with many proteins during its life cycle in cardiac cells, it would be of great interest to generate a mouse model that permits the biochemical purification of Na _v_1.5 with high efficiency, hence allowing the co-purification of interacting proteins. The identity of these co-purified proteins may then be determined by using mass spectrometry-based technologies. In order to do this, one needs to first generate a knock-in mouse model, where a high-affinity epitope would be added to the mouse *Scn5a* gene that codes for Na _v_1.5.

The goals of this short study were (1) to compare the biophysical properties of the sodium current (I _Na_) generated by mouse Na _v_1.5 and human Na _v_1.5 constructs expressed in HEK293 cells, and (2) to investigate the possible alterations of the biophysical properties of human Na _v_1.5 constructs that were modified with specific epitopes. We used the common fluorescent GFP and YFP proteins as epitopes, which provide the advantage of being detectable without the use of antibodies. However, these tags can only be added to the N- and C-termini, which are both intracellular in Na _v_1.5, and which are thus, not easily accessible. Therefore, we additionally chose the FLAG-epitope (Sigma-Aldrich), which consists of a short sequence that can be inserted into the extracellular loops of Na _v_1.5. The results of these studies will have to be taken into account when planning the generation of a mouse line bearing an epitope-tagged Na _v_1.5 channel.

## Methods

### Transfection and culture of HEK293 cells

HEK293 cells (Robert S Kass laboratory, Columbia University, New York) were transfected by Lipofectamine LTX (Invitrogen) according to the manufacturer‘s instructions. The plasmids used were the 2019 amino acid isoform of the mouse voltage-gated sodium channel (pcDNA3-mNa _v_1.5; a gift from Thomas Zimmer, University of Jena, Germany ^6^), human Na _v_1.5 (pcDNA3.1-hNa _v_1.5), and three differently tagged hNa _v_1.5 (pcDNA3.1-hNa _v_1.5-GFP-N-terminal, pEYFP-hNa _v_1.5, and pcDNA3.1-FLAG(299/300)-hNa _v_1.5). The FLAG-tag is an eight amino acid-long epitope (DYKDDDDK) that was inserted previously (by Robert S Kass laboratory) into the extracellular loop linking the transmembrane segments S5 to S6 of domain I, between the residues Leu-299 and Val-300; GFP and YFP were previously added by T. Zimmer to the N-terminal ^7^. For wild-type and tagged hNa _v_1.5, 1 µg of one of the listed plasmids, 1 µg of empty pcDNA3.1 (Invitrogen), and 0.4 µg of DNA coding for CD8 (Robert S Kass laboratory) were used for transfection. In order to measure currents of comparable size, 0.01–1 µg of mNa _v_1.5 was co-transfected with 1 µg of empty pcDNA3.1, and 0.4 µg of DNA coding for CD8. Transfected HEK293 cells were then grown in Dulbecco's modified Eagle's medium (DMEM) (Gibco) with 10% calf serum (Gibco), 0.2% glutamine (Sigma), and 20 mg/ml gentamycin (Gibco), and incubated at 37°C with 95%O _2_/5%CO _2_.

### Cellular electrophysiology

All experiments were performed in the whole-cell voltage-clamp mode. The extracellular solution contained (in mM): 50 NaCl, 80 NMDG-Cl, 5 CsCl, 2 CaCl _2_, 1.2 MgCl _2_, 10 HEPES, 5 Glucose, adjusted to pH 7.4 with CsOH, and with an osmolality of 280–290 mOsm. The internal solution consisted of (in mM): 70 CsAsp, 60 CsCl, 1 CaCl _2_, 1 MgCl _2_, 10 HEPES; 11 Cs _2_EGTA, 5 Na _2_ATP, adjusted to pH 7.2 with CsOH, and with an osmolality of 297 mOsm. Recordings were performed at room temperature (20–22°C) using a VE-2 amplifier (Alembic Instruments, Montreal, Canada). Data was acquired by Clampex 10.2 (Axon Instruments, Union City, Canada). Membrane resistance was ≥ 1 GΩ and access resistance ≤ 6.1 MΩ. Transfected cells were recognized by the addition of 1 µl/ml Dynabeads CD8 (Invitrogen) into the extracellular solution. Current-voltage (I/V) curves` were assessed by depolarising cells from a holding potential of -100 mV to voltages of between -80 and 40 mV during 20 ms. Steady-state inactivation properties were measured by the following protocol: the cells were kept at a holding potential of -100 mV and then hyper- and depolarised during 500 ms to voltages of between -120 and 0 mV in steps of 5 mV, followed by 20 ms at the voltage that elicited the maximal response during the I/V-protocol. Voltage-dependent activation was read either from the I/V- or the steady-state inactivation-protocol. To characterise the recovery from inactivation, the cells were depolarised from a holding potential of -100 mV for 100 ms, repolarised to -100 mV at a recovery time of 0.25–3000 ms, and depolarised again for 25 ms. By varying the time of the first depolarisation step from 3 to 3000 ms followed by 25 ms of repolarisation, the onset of slow inactivation was determined (see insets of Figure 2 and Figure 4).

### Data analyses and statistics

Peak values for all protocols were detected and measured by Clampfit 10.2 and I/V-relationships were fitted using KaleidaGraph 3.5 (Synergy Software, Reading, USA). Values were normalised to membrane capacitance. The following formula was used to fit I/V-curves and to calculate reversal potentials: I _Na_ = (G _max_(V-V _rev,Na_))/(1+e ^V-V0.5/K^) with I _Na_ = sodium current in pA, G _max_ = max. conductance = 60 Ω ^-1^, V _rev,Na_ = reversal potential = 40 mV, K = (-zδF)/FR = equilibrium constant = -5, V _0.5_ = voltage for 50% of maximum current = -20 mV. Activation and inactivation curves were fitted with the Boltzmann equation f _0_ = 1/(1+ e ^V-V0.5/K^) with f _0_ = fraction of open channels/total available channels. Statistical analyses were performed using two-tailed Student's t-tests. A p value <0.05 was considered statistically significant.

## Results

### Electrophysiological properties of human and mouse Na _v_1.5 are comparable

**Figure 1.**
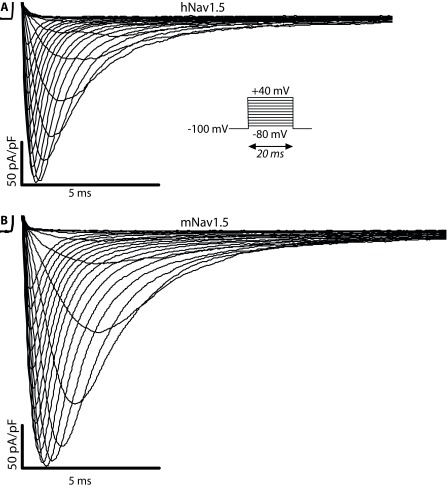
Representative I _Na_ recordings following the current voltage (I/V)-protocol described in the Methods. ( **A**) Voltage-dependent currents measured for hNa _v_1.5 expressed in a HEK293 cell. ( **B**) Data from the same protocol for mNa _v_1.5.

**Figure 2.**
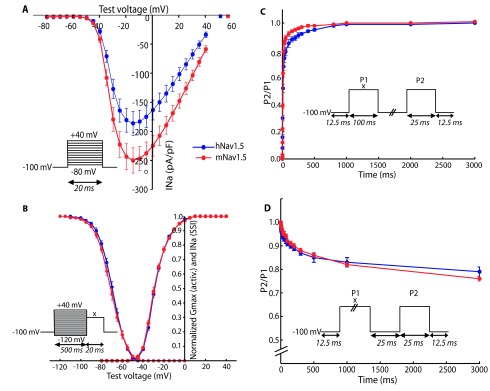
Electrophysiological properties of human and mouse Na _v_1.5. The voltage-clamp protocols used are shown in the corresponding insets. For **B–D**, the voltage x was adjusted to the voltage that elicited maximum current during the current voltage (I/V)-protocol (-10 to -30 mV). ( **A**) I/V-protocol for assessment of reversal potentials. Peak currents were measured for both channels at -15 mV. Calculated reversal potentials are marked with square data points. ( **B**) Voltage-dependence of activation and steady-state inactivation (SSI). The data was fitted with the Boltzmann formula. Only the slope of the inactivation curve differs between mouse and human sodium channels (shallower in mNa _v_1.5). ( **C**) Recovery from inactivation. The duration between the depolarising steps was varied from 0.25 to 3000 ms. mNa _v_1.5 had a slight tendency to recover faster than hNa _v_1.5. ( **D**) Onset of slow inactivation. The duration of the first step was varied from 0.25 to 3000 ms. The relative number of channels entering slow inactivation is similar for both types. ( **A–B**) n(hNa _v_1.5) = 22, n(mNa _v_1.5) = 17. ( **C–D**) n(hNa _v_1.5) = 9, n(mNa _v_1.5) = 7. **P<0.01 obtained by two-tailed Student's t-tests; error bars indicate standard errors.

To compare the biophysical properties of the cardiac sodium channel Na _v_1.5 from the human (hNa _v_1.5) or the mouse sequence (mNa _v_1.5), we measured the electrophysiological properties of hNa _v_1.5 and mNa _v_1.5, transiently expressed in HEK293 cells. Representative I _Na_ recordings are shown in Figure 1. The responses to all applied protocols revealed similar characteristics for both channels, except for the reversal potential and the slope of steady-state inactivation ( Figure 2 and Table 1). The peak currents from the I/V-protocol were at -15 mV for both channels ( Figure 2A). Furthermore, activation and inactivation of 50% of the channels occurred for both channels at ~-28 mV and ~-71 mV, respectively. In addition, the slopes of the activation curve were comparable for both channels (6.00 mV/e-fold in human and 6.24 mV/e-fold in mouse). Significant differences could be detected in the reversal potential V _rev_ (51.0 mV and 56.6 mV, P<0.01) and in the slope of the inactivation curve (5.95 mV/e-fold and 6.67 mV/e-fold, P<0.01) ( Figure 2B). In addition, mNa _v_1.5 had a tendency to recover faster from inactivation ( Figure 2C). The fraction of channels entering into a slow inactivation state was similar for both channel types ( Figure 2D).

**Table 1.** Summarized properties of human and mouse Na _v_1.5. Data was obtained with current voltage (I/V)- and steady-state inactivation protocols. Mean values and standard errors are shown. **P<0.01 obtained by two-tailed Student's t-tests.

		*hNa _v_1.5*	*mNa _v_1.5*
		mean ± sem	mean ± sem
*I/V*	V _rev_ (mV)	51.0 ± 0.9	**56.6 ± 0.8
*Activation*	V _1/2_ (mV)	-27.8 ± 0.5	-28.2 ± 0.7
	Slope (mV/e-fold)	6.00 ± 0.17	6.24 ± 0.16
*Inactivation*	V _1/2_ (mV)	-70.0 ± 1.0	-71.7 ± 1.1
	Slope (mV/e-fold)	5.95 ± 0.12	**6.67 ± 0.19
*Cell capacitance*	pF	16.0 ± 1.2	14.2 ± 0.7
*I _max_*	pA/pF	-185 ± 21	-249 ± 22
*n*		22	17

Raw data from sodium current recordings obtained from untagged human and mouse sodium channels (Nav1.5) expressed in HEK293 cells. For each channel type the cells are numbered starting from 1. For the sake of similar conditions (cells from the same passage, recordings on the same day, same solution aliquots), new cells have been recorded for the tag study. There are two sheets per channel type: one with data from the current voltage (I/V) activation, together with the steady-state inactivation (SSI) protocol; and a second one with data obtained with the onset of slow inactivation (OSI) and the recovery from inactivation (RFI) protocol. For some cells, only some of the protocols have been applied (thus non-continuous cell numbering in some OSI/RFI sheets). All measured voltages are given in mV; cell capacitance was measured in pF. Note that the cells transfected with untagged hNav1.5 used for comparison with mNav1.5 are not the same as in the study with different tags. See Figure 2 and 4 for information about the applied protocols.

### FLAG-tag inserted at the L299/V300 site alters voltage-dependent activation of hNa _v_1.5

**Figure 3.**
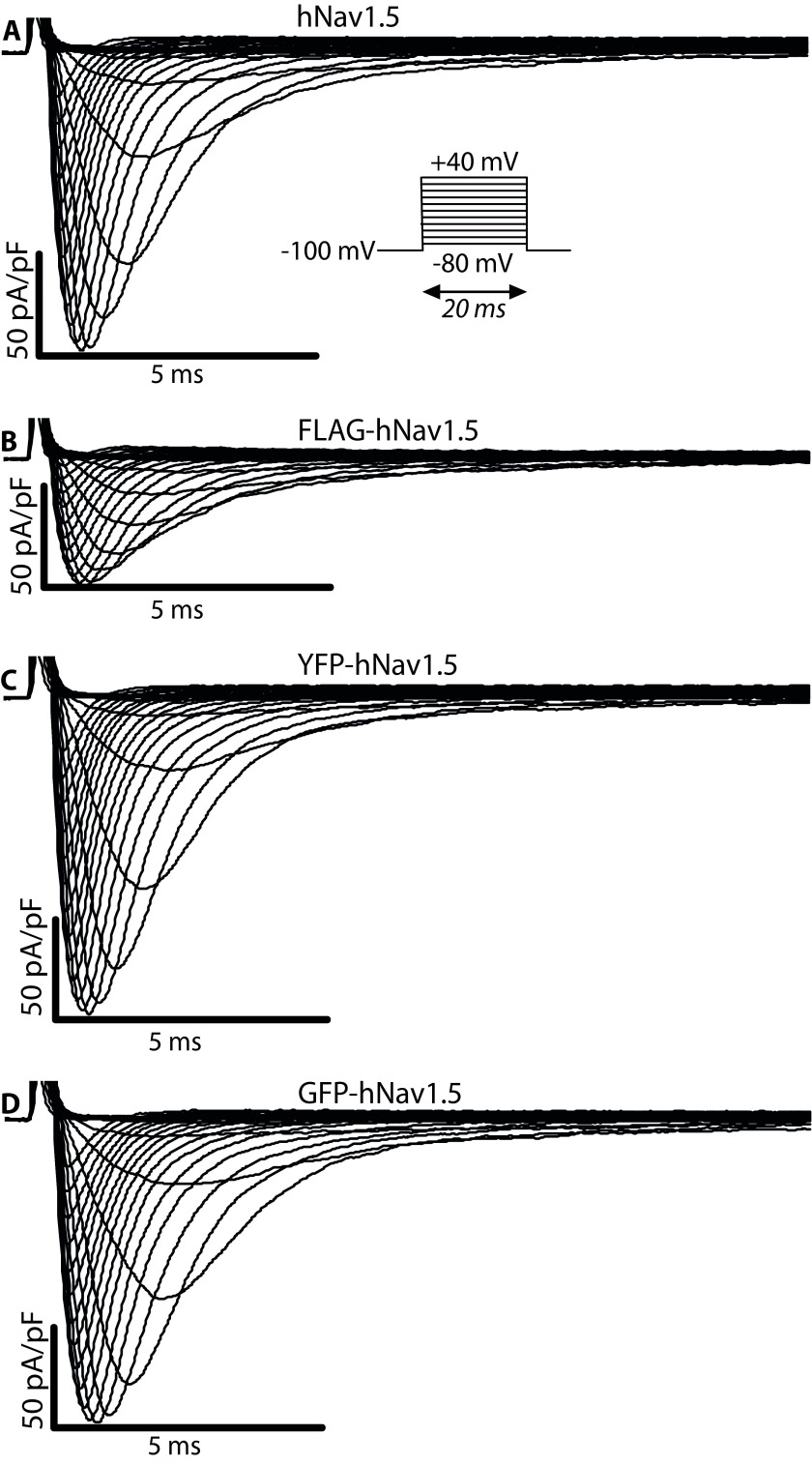
Representative sodium current (I _Na_) recordings. ( **A**) Voltage-dependent currents measured for hNa _v_1.5 expressed in a HEK293 cell. The same data for ( **B**) FLAG-hNa _v_1.5 ( **C**) YFP-hNa _v_1.5, and ( **D**) GFP-hNa _v_1.5.

**Figure 4.**
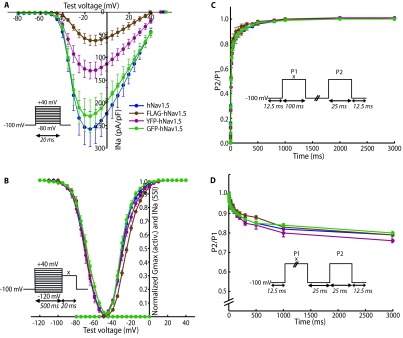
Electrophysiological properties of untagged and tagged hNa _v_1.5. The voltage-clamp protocols used are shown in the corresponding insets. For **B–D**, the voltage x was adjusted to the voltage that elicited maximum current during the current voltage (I/V)-protocol. ( **A**) I/V-protocol for assessment of reversal potentials. Tagging with N-terminal YFP and FLAG (L299/V300) significantly decreases peak currents. Calculated reversal potentials are marked with square data points. ( **B**) Voltage-dependence of activation and steady-state inactivation. The data was fitted with the Boltzmann formula. The activation slope of FLAG- and YFP-tagged channels is shallower compared to the untagged hNa _v_1.5. V _1/2_ is shifted by 5 mV for FLAG-hNa _v_1.5. ( **C**) Recovery from inactivation. The duration between the depolarising steps was varied from 0.25 to 3000 ms. No differences between the different channels could be detected. ( **D**) Onset of slow inactivation. The duration of the first step was varied from 0.25 to 3000 ms. The relative number of channels entering slow inactivation is similar for all four channel types. ( **A–B**): n(untagged) = 7, n(FLAG) = 11, n(YFP) = 11, n(GFP) = 8. ( **C**): n(untagged) = 12, n(FLAG) = 5, n(YFP) = 11, n(GFP) = 8. ( **D**): n(untagged) = 10, n(FLAG) = 8, n(YFP) = 10, n(GFP) = 8. **P<0.01 obtained by two-tailed Student's t-tests; error bars indicate standard errors.

The second set of experiments addressed the effects of adding epitopes to Na _v_1.5 on its biophysical properties. To do this, we assessed the influence of these epitopes on I _Na_ by expressing differently tagged hNa _v_1.5 in HEK293 cells and performing whole-cell voltage-clamp experiments similar to those described above. YFP- and GFP-tags were added to the N-terminus; the FLAG-tag was inserted into the extracellular loop linking S5 to S6 of domain I, between residues Leu-299 and Val-300. Representative I _Na_ recordings for all transfected constructs are shown in Figure 3 and the data is summarised in Table 2. With the exception of the GFP-tagged construct, tagging of hNa _v_1.5 led to a significant decrease in peak current I _max_ ( Figure 4A) compared to the control WT hNa _v_1.5 (FLAG: 57 pA/pF with P<0.01, YFP: 120 pA/pF with P<0.05, WT hNa _v_1.5: 240 pA/pF). Adding GFP did not affect any of the biophysical properties of the human sodium channel, while a shallower activation slope (6.87 vs. 5.91 mV/e-fold, P<0.05, Figure 4B and Table 2) was observed for the YFP-tagged channel. The most pronounced effects were observed for the FLAG-tagged hNa _v_1.5. The activation slope was significantly shallower (6.96 vs. 5.91 mV/e-fold, Figure 4B and Table 2), indicating that the activation of this channel is less sensitive to voltage changes. In addition, the V _1/2_ of activation was shifted towards more positive voltages by about 5 mV, with -23.9 mV in FLAG-hNa _v_1.5, compared to -28.9 mV in untagged hNa _v_1.5. Finally, the reversal potential was decreased in the FLAG-hNa _v_1.5 (FLAG 39.3 mV and untagged 51.8 mV, Figure 4B). Recovery from inactivation ( Figure 4C) and onset of slow inactivation ( Figure 4D) were comparable for all channels.

**Table 2.** Summarized properties of wild-type and tagged hNa _v_1.5. Data was obtained with current-voltage (I/V)-, and steady-state inactivation protocols. Mean values and standard errors are shown. *P<0.05, **P<0.01 obtained by two-tailed Student's t-tests (all statistics were calculated with untagged hNa _v_1.5 channel as a reference).

		*hNa _v_1.5*	*FLAG-hNa _v_1.5*	*YFP-hNa _v_1.5*	*GFP-hNa _v_1.5*
		mean ± sem	mean ± sem	mean ± sem	mean ± sem
*I/V*	V _rev_ (mV)	51.8 ± 0.9	**39.3 ± 2.2	49.1 ± 1.5	53.8 ± 1.9
*Activation*	V _1/2_ (mV)	-28.9 ± 0.6	**-23.9 ± 0.5	-27.5 ± 1.1	-29.8 ± 1.1
	Slope (mV/e-fold)	5.91 ± 0.17	**6.96 ± 0.14	*6.87 ± 0.27	5.40 ± 0.36
*Inactivation*	V _1/2_ (mV)	-70.6 ± 1.2	-70.0 ± 1.1	-71.2 ± 1.4	-68.9 ± 1.2
	Slope (mV/e-fold)	5.95 ± 0.24	5.33 ± 0.15	5.69 ± 0.19	6.37 ± 0.19
*Cell capacitance*	pF	14.4 ± 1.6	16.3 ± 1.1	14.5 ± 0.9	14.0 ± 0.8
*I _max_*	pA/pF	240 ± 36	**57 ± 11	*120 ± 17	214 ± 33
*n*		7	11	11	8

Raw data from sodium current recordings obtained from tagged human sodium channels (Nav1.5) expressed in HEK293 cells. For each channel type the cells are numbered starting from 1. For the sake of similar conditions (cells from the same passage, recordings on the same day, same solution aliquots), new cells have been recorded for the tag study. There are two sheets per channel type: one with data from the current voltage (I/V) activation, together with the steady-state inactivation (SSI) protocol; and a second one with data obtained with the onset of slow inactivation (OSI) and the recovery from inactivation (RFI) protocol. For some cells, only some of the protocols have been applied (thus non-continuous cell numbering in some OSI/RFI sheets). All measured voltages are given in mV; cell capacitance was measured in pF. Note that the cells transfected with untagged hNav1.5 used for comparison with mNav1.5 are not the same as in the study with different tags. See Figure 2 and 4 for information about the applied protocols.

## Discussion

The present study demonstrates (1) that the biophysical properties of mouse Na _v_1.5 are essentially similar to the human homolog when expressed in HEK293 cells, and (2) that adding epitopes either upstream of the N-terminus of human Na _v_1.5 or in one of the extracellular loops reduces the amount of I _Na_ and alters some of its biophysical properties. Interestingly, GFP in the N-terminus was the only epitope that did not modify any of the measured biophysical properties of hNa _v_1.5. The most pronounced effects could be observed by the insertion of the FLAG-tag in an extracellular loop. In this construct, not only was the amount of I _Na_ drastically decreased, but also the activation properties of the sodium channel were altered. The smaller changes found in the properties of YFP-hNa _v_1.5 might be partially linked to the different vector used for this epitope, especially since no alterations could be observed for GFP-hNa _v_1.5.

The limitations of studying mutant Na _v_1.5 channels in mammalian cells have been demonstrated in two recent studies. First, Mohler and colleagues ^8^ observed that the Brugada syndrome causing mutant p.E1053K Na _v_1.5 channel did not display any trafficking defect in HEK293 cells, while it failed to traffic to the intercalated discs when expressed in rat ventricular cells. Second, the Na _v_1.5 p.D1275N variant, found in patients with dilated cardiomyopathy and various arrhythmias and conduction disease, was also found to display reduced expression in knocked-in mouse cardiac tissue and defective expression at the lateral membrane of ventricular myocytes ^9^. However, when expressed in chinese ovary cells, the p.D1275N variant had properties that were undistinguishable from wt channels. These observations demonstrate that, while useful to study their intrinsic biophysical properties, the mammalian cells that are used as expression systems have clear limitations when studying the trafficking properties of ion channels. Generation of genetically-modified animal models is one of the most powerful, albeit time-consuming, approaches.

However the findings of the present study have to be taken into account when planning to generate such mouse models that harbour specific epitopes in the mouse Na _v_1.5 gene. Different combinations of epitopes and insertion sites might reveal better candidates for *in-vivo* approaches. Furthermore, additional studies should be performed in HEK293 cells co-expressing other subunits and regulating proteins, and in native cardiomyocytes in order to assess the effects of added epitopes on the interactions with these proteins.
