# Supplementary material for: Electrophysiological properties of mouse and epitope-tagged human cardiac sodium channel Na v1.5 expressed in HEK293 cells
Source: F1000Res. 2013 Apr 5;2:48. Originally published 2013 Feb 13. [Version 2] doi: 10.12688/f1000research.2-48.v2 (PMC3869486; doi:10.12688/f1000research.2-48.v2)
Supplement: Peer review history

# Peer review: Referee response for version 2

**DOI:** 10.5256/f1000research.1309.r3189

**Published:** 2014-01-20

**Authors:** Céline Fiset

**Affiliations:** 1 Faculty of Pharmacy, Centre de Recherche de l’Institut de Cardiologie de Montréal, Université de Montréal, Montréal, QC, Canada

I have read this submission. I believe that I have an appropriate level of expertise to confirm that it is of an acceptable scientific standard.

# Peer review: Referee response for version 2

**DOI:** 10.5256/f1000research.1309.r958

**Published:** 2013-05-20

**Authors:** Jamie Vandenberg

**Affiliations:** 1 Division of Molecular Cardiology and Biophysics, Victor Chang Cardiac Research Institute, Darlinghurst, NSW, Australia

The discussion has been appropriately modified to take into account the suggestions I made after reviewing the first version of the manuscript. The authors have not however added in any more data or analysis of the trafficking phenotypes of the tagged proteins. I still believe that data comparing the forward trafficking and stability of the different epitope tagged constructs would be worth having. This could be achieved using Western blot / immunohistochemistry and/or live cell imaging.

I have read this submission. I believe that I have an appropriate level of expertise to confirm that it is of an acceptable scientific standard.

# Peer review: Referee response for version 1

**DOI:** 10.5256/f1000research.1119.r798

**Published:** 2013-02-25

**Authors:** Jamie Vandenberg

**Affiliations:** 1 Division of Molecular Cardiology and Biophysics, Victor Chang Cardiac Research Institute, Darlinghurst, NSW, Australia

This manuscript from Reinhard and colleagues describes the electrophysiology of epitope tagged sodium channels expressed in mammalian cell lines. This system is commonly used for the characterizing the electrophysiological and/or trafficking phenotypes of clinically occurring mutants. Some of the constructs described in this study have been reported before (see e.g. reference 7 in the manuscript: Zimmer T, Biskup C, Dugarmaa S, *et al.*: Functional expression of GFP-linked human heart sodium channel (hH1) and subcellular localization of the a subunit in HEK293 cells and dog cardiac myocytes. *J Membr Biol*. 2002; **186 **(1): 1–12) but to my knowledge the construct with the insertion of a FLAG-tag into the extracellular loop linking S5 to S6 of domain I, between residues Leu-299 and Val-300, is novel. Whilst it is unfortunate that this construct has altered electrophysiological properties, it should be useful for live cell trafficking assays as the extracellular epitope can be recognized without having to lyse the cells. Reagents such as those described in this study have been extremely useful and will continue to be so. In this context it is useful to have proper baseline characterization of their properties, which can be used as a reference point for other users. 

The manuscript however could have benefited from a more thorough analysis of the trafficking properties of the tagged channels and a broader discussion of the advantages and limitations of the use of the mammalian expression system for characterizing mutant sodium channels. One particularly pertinent recent example of the limitations of the heterologous expression system is that of the D1275N cardiac sodium channel which causes minimal perturbation to gating when expressed *in vitro* but has a marked loss of function when expressed *in vivo* in gene-targeted mice  (see Watanabe H *et al.*, Striking In Vivo Phenotype of a Disease-Associated Human SCN5A Mutation Producing Minimal Changes in Vitro, *Circulation*. 2011; **124**:1001-1011.)

I have read this submission. I believe that I have an appropriate level of expertise to confirm that it is of an acceptable scientific standard, however I have significant reservations, as outlined above.

# Peer review: Referee response for version 1

**DOI:** 10.5256/f1000research.1119.r777

**Published:** 2013-02-18

**Authors:** Céline Fiset

**Affiliations:** 1 Faculty of Pharmacy, Centre de Recherche de l’Institut de Cardiologie de Montréal, Université de Montréal, Montréal, QC, Canada

I have read this submission. I believe that I have an appropriate level of expertise to confirm that it is of an acceptable scientific standard.
